# Effect of Receiving Text Messages on Health Care Behavior and State Anxiety of Thai Pregnant Women: A Randomized Controlled Trial

**DOI:** 10.30476/IJCBNM.2021.89364.1604

**Published:** 2022-01

**Authors:** Piyanut Xuto, Kodama Toyohiko, Piyaporn Prasitwattanaseree, Punpilai Sriarporn

**Affiliations:** 1 Department of Obstetrics and Gynaecology, Faculty of Nursing, Chiang Mai University, Chiang Mai, Thailand; 2 Department of Nursing, School of Health Science, University of Occupational and Environmental Health, Fukuoka, Japan

**Keywords:** Anxiety, Health care behavior, Pregnant woman, Text messages

## Abstract

**Background:**

The health care behavior of Thai pregnant women should enhance. Pregnant women are facing anxiety from a variety of issues. Current evidence suggests that a text message can support health care services and reduce anxiety. This study aimed to examine receiving text messages on health care behavior and state anxiety among Thai pregnant women.

**Methods:**

This study was a single-blind randomized controlled trial. A sample of 66 primigravidas was randomly assigned using a random number table, 33 per group. All participants received a recommendation for healthy behavior during pregnancy. The intervention group added 56 text messages between 13 and 40 gestational weeks, from two government hospitals in Thailand, from March 2018 to May 2019. The data collection tool comprised of demographic characteristics questionnaire, Pregnancy Outcomes Record, The Health Care Behavior during Pregnancy Questionnaire (HCBPQ) (Thai version) which developed by the researcher, and State-Trait Anxiety Inventory (STAI-S). Data were analyzed via SPSS version 18 using descriptive statistics, independent t-test, Fisher’s exact test, and chi-square. The significance level was considered P<0.05.

**Results:**

The results showed that the intervention group adopted appropriate mean health care behaviors significantly only in physical activity domain (15.40±3.19) compared to the control group (13.58±1.89), (P=0.01) and revealed a significantly lower total mean score of state-anxiety than the control group (35.23±8.50 vs. 40.79±9.28, P=0.02). Other health care behavior domains between the two groups were not statistically significant (P>0.05).

**Conclusion:**

Text messages could increase physical activity in Thai pregnant women and reduce the total score of anxiety during pregnancy. Thus, the text message strategy is appropriate to use during the antenatal period.

**Trial Registration Number:**

TCTR20180814005

## INTRODUCTION

Text messages or short message services (SMS) are a type of mobile health (mHealth) service that refers to the mobile technology used to support health care services. ^1^ Promoting women’s physical and mental health during pregnancy is necessary and should be undertaken continuously. Pregnant woman can achieve health promotion in several ways, such as developing health literacy, building a healthy setting, or strengthening health promotion capacity and personal skills. Systematic reviews of the literature reveal that SMS intervention encourages health-promoting behaviors among pregnant women. ^2 - 4^ Based on the systematic review, this study aims to assess the effect of mHealth interventions in improving maternal health. ^5^ They conducted a Strength, Weakness, Opportunity, and Threat analysis of 27 studies and found strengths of accessibility including elements such as providing easy to understand messages, low costs for user and implementer, and applicability to rural areas. However, they also found weaknesses of usability that still require rectification, such as text limits that need full-text designs to convey health messages within the limit effectively and inadequate durability of phones. Furthermore, although SMS targeted at pregnant women is an effective method to increase and promote health care behaviors during pregnancy, strategies to improve SMS implementation in Antenatal Care (ANC) need to be explored. ^5^

SMS functions can be classified into five categories: education, monitoring, reminder, communication and support, and emergency medical response system. ^5 - 7^ Based on a systematic review, education is the most popular SMS function and comprises at least 80% of all parts. ^5^ Additionally, it can remind the individuals to repeat their behaviors. Self-monitoring, the critical concept of mobile messages affecting health conditions, consists of three elements based on the concept of Coomes: 1) frequency, 2) time period, and 3) tailored for a pregnant woman. ^8^ To increase health-promotion behaviors and reduce usability weaknesses, messages should be sent twice a week, between 13 weeks of gestation to the end of the pregnancy. Sending the messages allows women to use such information to adjust their behaviors by reducing risky behaviors and developing appropriate health-promoting behaviors. In addition, sending a warning message or birth preparedness can help pregnant women to modify behavior and their attitude. ^7^

Moreover, when these messages are tailored for pregnant women, they seem specific to their conditions. For example, increased weight of the fetus, placenta, and uterus causes pregnant women to lean forward while walking, inducing back pain. Sending customized messages helps pregnant women understand how to relieve such discomfort. Hence, the three elements of frequency, time period, and tailored directly reduce risky behaviors and promote appropriate self-care behaviors. Moreover, during pregnancy, primigravidae have higher antenatal anxiety than multigravidas. Approximately 24% of primigravidae report increased anxiety levels such as unplanned pregnancy, high financial stressors, and low social support. ^9^ Maternal anxiety during pregnancy can affect poor pregnancy outcomes such as preterm birth, low birth weight, small for gestational age, and quality of life of pregnant women. ^10 - 12^ Furthermore, antenatal anxiety still affects the newborns’ health which is classified into four groups: biological, mental, behavioral, and medical. ^13^ Therefore, receiving tailored text messages might reduce the anxiety experienced during the prenatal period. ^14^ Moreover, SMS was introduced to promote health to achieve the highest health outcome, health promotion, and disease prevention during pregnancy. Thus, this study examines the health care behaviors and state anxiety among Thai women in the intervention and control groups. 

## MATERIALS AND METHODS

This study was a single-blind randomized controlled trial. Sixty-six participants were pregnant women who visited ANC clinic of two government hospitals in Chiang Mai and Ubon Ratchathani provinces of Thailand from March 2018 to May 2019.

For sample size calculation, power analysis used at .80 had a statistically significant level of 0.05. The effect size was modeled on previous studies that resemble the current research on anxiety and pregnancy outcomes in obtaining messages through mobile phones. ^14^ For statistical superiority design, ^15^ the formula was as follows:

$N=2{\left(\frac{Z_{1-\alpha /2}+Z_{1-\beta }}{\delta }\right)}^{2}xS^{2}$

*N*=size per group; *δ*=the real difference between two treatment effect; *S*=standard deviation of comparison group; 

$N=2{\left(\frac{1.96+0.845}{2.15}\right)}^{2}x2.89^{2}$

*N*=28.447

The desired sample size was decided 28 participants per group. The sample size assumed a 20% attrition rate combined with one year. The number of participants in this study was 66 (33 in each group). 44 participants were enrolled from the Maharaj Nakorn Chiang Mai Hospital, Chiang Mai, and 22 from the Sunpasitthiprasong Hospital, Ubon Ratchathani.

The study recruited pregnant women meeting the inclusion criteria for the study at the end of their initial prenatal care visit. The inclusion criteria were primigravida and singleton pregnancy, gestational period of 12±1 week ascertained by the last menstrual period date, age between 18 and 40 years, low-risk pregnant women not suffering from any chronic diseases or having an experience of infertility problems, possession of a smartphone that can receive SMS. Following the ANC process, the research team asked the women if they were willing to participate in the study. Those who agreed were asked to supply their written informed consent and complete the demographic characteristics Questionnaire. Individuals who needed more time to decide whether they wanted to participate could think and provide a final decision by texting back to the research team. The exclusion criteria were any condition that would limit the ability to use the benefits of text messages, such as twin pregnancy, and the women unwilling to participate in the study due to threatened abortion or termination of pregnancy.

**Figure 1:**
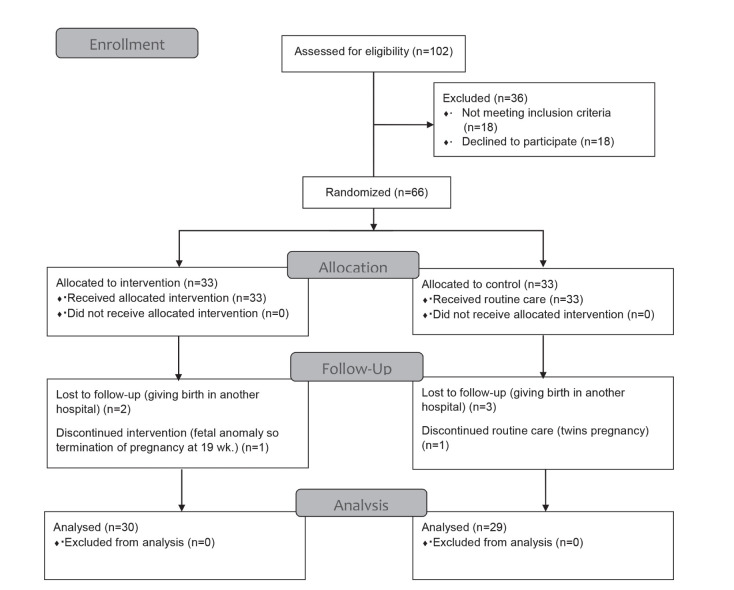
CONSORT flow chart of the participants

The number of participants eligible for the study was 102 subjects. Thirty-six subjects were excluded from the study, and finally 66 subjects were randomly categorized into two groups by using a random number table (Figure 1). The table was developed from computer-generated random numbers, 1=A, 2=B, 3=B, 4=A, until 44 (A=22, and B=22). Group A was the intervention group, whereas group B was the control group. Each setting assigned the participants into both group sequences; 22 and 11 participants were enrolled, respectively, in the intervention and control groups. The randomization procedure was performed outside the actual clinical setting. Each participant was assigned a participant ID. Clinicians who delivered healthcare services at the clinics were single blinded to the intervention. The participants in both groups received ANC services, antenatal screening tests, maternity information to have a healthy pregnancy from the nurses as usual. In addition, the intervention group received text messages via smartphones during 13-40 gestational weeks every Monday and Thursday at 10 am.

Fifty-six messages were developed. The content of the messages corresponded to the physiological changes experienced during pregnancy. The themes of the text messages comprised nutrition, coping with discomfort during pregnancy, warning signs of pregnancy complications, mental health care, preparation for experiencing labor, and signs and symptoms that lead to the delivery stage. For example, at week 19, the participants received the following message: “You can get gestational anemia easily. Please have a diet that includes iron, red meat, fish, green vegetables, and tofu. Moreover, vitamin C can promote iron absorption. Therefore, eat the five food groups.” All the statements were evaluated by a panel of three experts, including health education, maternal and child health nursing, and midwifery specialists who evaluated the scale for validity. After the modifications provided by the experts, the content validity index was equal to 1.

Data collection tools included: 1) Demographic characteristics Questionnaire, which documented the participants’ age, education level, occupation, pre-pregnant weight, pre-pregnancy body mass index (BMI), income, and the contact channel to be used to inform the researchers when they go into labor. 

2) Pregnancy Outcomes Record was composed of weight gain throughout pregnancy, blood sugar testing, hemoglobin, baby weight, type of delivery, and complications during pregnancy and labor, such as a gestational diabetes mellitus, pregnancy-induced hypertension, and anemia, as obtained from medical records. For laboratory reliability, two laboratories in the hospitals rely on the standard protocol, international organization for standardization (ISO) 15189, which is specific to medical laboratories. 

3) The Health Care Behavior during Pregnancy Questionnaire (HCBPQ) (Thai version), a 33-item scale, developed by the researchers’ team to assess the participants’ behaviors. It is scored based on a 5-point Likert scale, from “never done” (1) to “always done” (5). The 3-expert panel (two nursing instructors who supervised the antenatal clinic, and one obstetrician) evaluated the questionnaire as above. After modifications provided by the experts, the Content Validity Index was equal to 1. The construct validity by confirmatory factor analysis was applied. Five components were tested: nutrition (items 1-11), physical activity (items 12-15), healthy behaviors (items 16-25), sign & symptoms management (items 26-30), and labor practice (items 31-33). Each domain was scored by summing the items of each domain. The total score was obtained by summing all the subscale scores. Scores of this scale ranged from 33-165 (the minimum and maximum of each domain: nutrition 11-55; physical activity 4-20; healthy behaviors 10-50; sign & symptoms management 5-25; and labor practice 3-15. High scores indicate better healthy behaviors during pregnancy. The construct validity revealed the goodness of fit (chi-square=44.23, df=35, P=0.136, RMSEA=0.06, CFI=0.94, AGFI=0.92, GFI=0.91). All components had a moderate value of internal consistency using alpha coefficient of 0.689-0.855, and reliability was 0.80. using Cronbach’s alpha 

4) State-Trait Anxiety Inventory (STAI) which was developed by Spielberger in the year 1970, ^16^ was used. The STAI is composed of two 20-items for state and trait anxiety. Regarding the characteristic of each anxiety type, trait anxiety is affected by personality features and state anxiety intense from emotional features; thus, this study used only state anxiety measurement (STAI-S), the reliability and validity of which were checked by Delgado et al. Twenty items are rated on a 4-point Likert scale, from “almost never” (1) to “almost always” (4); the scores range from 20-80. Higher scores indicate greater anxiety for psychometric properties of state anxiety measurement in the term pregnancy population. ^17^ There were two factors; both factors had an appropriate value of internal consistency using alpha coefficient of 0.723-0.830. The first factor, i.e. “absence of anxiety”, consists of 10 items (items 1,2,5,8,10,11,15,16,19, and 20). In the analysis, the recoding of factor 1 was applied. The second factor, i.e. “presence of anxiety”, consists of 9 items (items 3,6,7,9,12,13,14,17, and 18). Item 4 (“I regret it”) did not have the representative value, and it was excluded from the factor 2. With the removal of item 4, the score changed from 19 to 76 points. The instrument used in this study was the Thai version, the reliability of which has been reported 0.89 using Cronbach’s alpha ^18^ the reliability obtained in this study was 0.83. 

The participants who were randomly assigned to the intervention group received text messages for 28 weeks. The text messages were sent from the automatic SMS program, SMS4P version 1.0. The program was designed and authorized by the Chiang Mai University, under the supervision of Dr. Kodama Toyohiko. The researcher was trained in using the SMS4P program before sending the first message. Once enrolled, the participants received welcome and introductory messages and began receiving two messages per week, and eventually received a total of 56 messages. If the participants chose the termination option, they were disenrolled from the study. Table 1 shows the issue of text messages appropriate to physiological and mental changes during the antenatal period. Text messages were composed of nutrition, coping with discomfort during pregnancy, warning signs of pregnancy complications, mental health care, preparation for experiencing labor, and signs and symptoms that lead to the delivery stage.

**Table 1:** Issue of text message during 13-40 gestational weeks

Week	Issue of text messages	Week	Issue of text messages
13 (1)	Thank you for participating in our study	27 (1)	Prenatal depression
13 (2)	Introduction of consultation services	27 (2)	Preventing prenatal depression
14 (1)	Baby’s development and introduction of the dietary for a pregnant woman	28 (1)	Fetal movement count
14 (2)	Avoid smoking, second smoking, and other air pollution	28 (2)	Sciatica pain during pregnancy
15 (1)	Changes in appetite and weight increases	29 (1)	Introduction of childbirth class
15 (2)	Preventative from constipation	29 (2)	Kegel exercise
16 (1)	Preventive from asymptomatic urinary infection	30 (1)	Heartburn during pregnancy
16 (2)	Oral health care promotion	30 (2)	Bonding with the baby
17 (1)	Preparing for a special investigation	31 (1)	Edema and care
17 (2)	The goal of gestational weight gain	31 (2)	Dyspnea during pregnancy
18 (1)	The choice for underwear fitting	32 (1)	Gestational weight gain
18 (2)	Baby growth and movement	32 (2)	Colostrum
19 (1)	Partner’s relationship	33 (1)	Urinary frequency and thirst
19 (2)	Prevention of gestational anemia (iron deficiency anemia)	33 (2)	Pruritic Urticarial Papules and Plaques ofPregnancy
20 (1)	Preventing premature birth	34 (1)	Minor discomfort during pregnancy
20 (2)	Physical activity appropriates the body condition.	34 (2)	Preparing before baby arrives
21 (1)	Prevent constipation	35 (1)	Mindful of vaginal discharge
21 (2)	Prevent pregnancy hypertension syndrome	35 (2)	Continuous fetal movement count
22 (1)	Prepare for breastfeeding	36 (1)	False or True labor pain
22 (2)	If you have hip joint pain	36 (2)	Time to go to the hospital
23 (1)	Prevent dry, cracked, itchy skin	37 (1)	Lightening
23 (2)	Prevent of melasma	37 (2)	Preparing for labor
24 (1)	Checking of GCT^a^	38 (1)	Ways to cope with labor pain_1
24 (2)	Prepare for OGTT^b^, incase	38 (2)	Ways to cope with labor pain_2
25 (1)	Prevention of poor circulation in legs	39 (1)	What to bring to the labor
25 (2)	Safe sex position during pregnancy	39 (2)	Notice the body
26 (1)	Leg cramp during pregnancy	40 (1)	Relax body and mind
26 (2)	Avoid fall during pregnancy	40 (2)	Notify message delivery end

a Glucose Challenge Test,

b Oral Glucose Tolerance Test

Before the data collection began, the research team held an introductory meeting with the staff of the ANC clinics. All participants received ANC service, screening risks in pregnancy, and health care recommendations. When the women gave birth to their babies, they contacted the research team through an informed channel like Line, Facebook, or email. The research team recorded the pregnancy outcome from the medical record. The participants were asked to complete the paper-based HCBPQ and the STAI-S questionnaire.

SPSS, version 18, was used in all analyses. Descriptive statistics were calculated for all outcomes and demographic characteristics. Cross-tabulations of the data between groups were performed; t-test, the Fisher’s Exact test, and chi-square test were used. Health care behaviors and state anxiety between the intervention and control groups were computed using t-test. The significance level was considered P<0.05.

The study protocol was approved as minimal risk research by the Faculty of Nursing, Chiang Mai University Institutional Review Board (Project No. 125-2017), and approved by the Institute Review Board of the Faculty of Medicine, Chiang Mai University (study code: NONE-2018-05406). Additionally, the study protocol also received the Ethics Certificate of Research Project in Humans from Sunpasitthiprasong Hospital, Ubon Ratchathani; Certificate Code 079/2017.

## RESULTS

Of the 66 women who entered the study, 59 (91%) completed the 28-week research (30 (90.91%) and 29 (87.88%) women in the intervention and the control groups, respectively). The participants were not statistically different in age, pre-pregnancy weight, pre-pregnancy BMI, education, occupation, and income (P>0.05). The intervention group had a mean age of 28.10±4.90 years, mean pre-pregnancy weight of 53.34±9.25 kg, and mean pre-pregnancy BMI of 20.95±3.35 kg/m^2^; their average income was 448.36±137.19 USD. The control group had a mean age 27.55±5.39 years, mean pre-pregnancy weight of 57.49±10.73 kg, and mean pre-pregnancy BMI of 22.33±3.58 kg/m^2^; their average income was 447.39±132.30 USD. 

As shown in Table 2, testing the differences between demographic characteristics of the two groups using Fisher’s Exact test showed no differences in either group for the variables. 

**Table 2:** Comparison of demographic characteristics between the groups at baseline

Variables	Intervention (N=30) N (%)	Control (N=29) N (%)	P value*
Education			
Diploma or lower	10 (33.33)	17 (58.62)	0.06
Bachelor or higher	20 (66.67)	12 (41.38)	
Pre-pregnancy BMI^a^ (kg/m^2^)			
Underweight	5 (16.67)	5 (17.24)	0.32
Normal	22 (73.33)	17 (58.62)	
Overweight	3 (10.00)	7 (24.14)	
Occupation			
Employee	18 (60.00)	18 (62.07)	0.76
Government	7 (23.33)	8 (27.59)	
Housewife	5 (16.67)	3 (10.34)	
Income (USD)^b^ per month			
<300	5 (16.66)	3 (10.34)	0.39
300-600	19 (63.33)	23 (79.31)	
>600	6 (20.00)	3 (10.34)	

* Fisher’s Exact Test;

a Body Mass Index;

b 1 USD=31.40 Baht

Post-intervention testing was conducted between the intervention and control groups; the results showed that the state-anxiety, both factor 1, 2, and the total score of the control group were higher than those of the intervention groups. However, only factor 2 and the total score of STAI-S had a statistically significant difference between the two groups. The results found no other aspect with a statistically significant difference between the intervention and control groups (Table 3). The number of people with pregnancy complications was 6 (20.00%) in intervention and 9 (31.03%) in the control groups, respectively.

**Table 3:** Comparison of pregnancy outcomes and state anxiety between the groups after the intervention

Factors	Intervention (N=30)	Control (N=29)	P value
	Mean±SD	Mean±SD	
Gestational weight gain (kg)	12.77±4.92	11.98±6.42	0.60*
Gestational age at birth (wk)	38.40±1.04	37.90±1.65	0.16*
Hemoglobin (gr/dl)	12.44±1.25	11.75±1.37	0.33*
GCT^a^ (mg/dl)	130.27±28.31	141.90±13.11	0.12*
Baby weight (kg)	2.93±3.65	2.96±4.46	0.73*
State-anxiety			
Factor 1	17.56±5.26	19.20±4.72	0.21*
Factor 2	16.30±4.97	19.68±4.95	0.01*
Total	35.23±8.50	40.79±9.28	0.02*
	N (%)	N (%)	
Gestational weight gain€ (kg) Underweight groups			
12.7-18.1	2 (6.67)	0 (0.00)	0.44**
>18.1	3 (10.00)	5 (17.24)	
Normal			
11.3-15.9	10 (33.33)	4 (13.79)	0.19**
>15.9	12 (40.00)	13 (44.83)	
Overweight			
6.8-11.3	1 (3.33)	1 (3.45)	1.00**
>11.3	2 (6.67)	6 (20.69)	
Pregnancy complications			
GDM^b^	4 (13.33)	8 (27.58)	
PIH^c^	2 (6.67)	1 (3.45)	
Anemia	0 (0)	0 (0)	
NO complication	24 (80)	20 (68.97)	0.33***
Birth type			
Natural Vaginal Delivery	18 (60.00)	19 (65.52)	0.66***
Elective Cesarean	12 (40.00)	10 (34.48)	

* Independent t-test;

** Fisher’s Exact Test;

*** Chi-square test;

€ Category based on IOM19;

a Glucose challenge test;

b Gestational Diabetes Mellitus;

c Pregnancy-Induced Hypertension

Table 4 reveals that the physical activity domain of health care behavior between the groups was significantly different. And five items showed that the pregnant women of the intervention group had a better score than the control group, such as “I drink fruit juice” and “I abstain from sexual intercourse for longer than a month” (P<0.05).

**Table 4:** Comparison of health care behavior scores of each item between the groups after the intervention

Items	Intervention (N=30)	Control (N=29)	P value*
	Mean±SD	Mean±SD	
**Nutrition**	38.26±3.95	38.96±3.58	0.48
1. I eat a variety of vegetables	4.40±0.77	4.28±0.75	0.43
2. I eat a variety of fruits	4.37±0.72	4.52±0.63	0.42
3. I eat fried and unhealthy food#	2.67±0.92	2.69±0.71	0.52
4. I eat sweets and desserts#	2.53±1.14	2.79±1.05	0.26
5. I eat pork, chicken, or fish for every meal	3.93±0.74	4.14±0.69	0.27
6. I drink 2 glasses of milk every day	4.73±0.52	4.48±0.87	0.36
7. I drink fruit juice#	3.37±0.96	3.86±0.88	0.04
8. When I am hungry, I eat anything that I want#	3.47±1.01	3.41±0.95	0.56
9. I eat grilled food which is rare to medium rare#	1.47±0.63	1.72±0.84	0.27
10. I take the vitamins prescribed by the hospital	4.93±0.25	4.69±0.66	0.10
11. I take herbs	2.40±1.59	2.38±1.21	0.86
**Physical activity**	15.40±3.19	13.58±1.89	0.01
12. I exercise by walking	3.97±1.13	4.03±0.78	0.88
13. I stretch my body	3.50±1.36	2.86±0.79	0.02
14. I squeeze and ease the muscle when my leg cramps	4.17±0.95	3.03±1.30	0.001
15. I try to sit and stand in a straight posture	3.77±1.01	3.66±0.94	0.41
**Healthy behaviour**	37.46±6.57	37.10±5.27	0.81
16. I take a short nap in the daytime	3.23±1.61	3.17±1.20	0.73
17. I brush my teeth after meals	3.67±1.21	3.79±0.94	0.89
18. I talk to the baby when it is kicking	4.37±0.67	4.31±0.76	0.87
19. I abstain from sexual intercourse for longer than a month#	4.03±1.25	4.62±0.98	0.01
20. I regularly defecate	4.17±0.75	3.55±0.78	0.004
21.When I do not want to pee frequently, I reduce my water consumption#	2.87±1.46	2.45±1.09	0.28
22. I go to the restroom when I need to pee strongly#	2.30±1.12	2.59±0.95	0.24
23. From 7 months, I intend to count fetal movements as suggested	4.47±0.51	4.38±0.82	0.93
24. I try to avoid stress and keep a clear mind	4.37±0.93	4.10±0.90	0.16
25. I know the emergency number of the hospital that I need to go to	4.00±1.05	4.14±1.16	0.49
**Sign & Symptoms management**	17.56±3.13	17.41±2.66	0.84
26. I often notice whether my urine is dark or opaque	4.10±0.85	4.17±0.85	0.73
27. In case I have leucorrhea, I keep my external reproductive organs clean	4.30±0.84	3.97±0.91	0.14
28. I notice swelling in my upper ankle (s), face, and hand (s)	4.33±0.61	4.34±0.55	1.000
29. In case the fetal movement count is less than ten until noon, I will wait to count the next day again #	2.67±1.49	2.28±1.33	0.28
30. In case the amniotic bag breaks but with no accompanying pain, I will wait for another day#	2.17±1.26	2.66±1.40	0.15
**Labor practice**	13.16±1.64	13.13±1.57	0.94
31. I will go to the hospital immediately at the onset of labor pain#	4.30±0.95	4.10±0.90	0.33
32. I am trained in how to control labor pains	4.00±0.95	4.17±0.81	0.50
33. I am trained in how to push during labor	4.87±0.35	4.86±0.35	0.95
**Total**	121.86±12.96	120.20±9.50	0.57

* Independent :t-test;

# Negative items

## DISCUSSION

This study showed that text messages could increase physical activity in Thai pregnant women and reduce the total score of anxiety during pregnancy. The messages in this study were a message to remind pregnant women to have a healthy behavior in terms of nutrition and physical activity, cope with discomfort during pregnancy, be alert to warning signs of pregnancy complications, support mental health care, and prepare for giving birth. After the intervention was completed, the results showed that health care behaviors significantly differed between the groups in only one domain, physical activity. As many studies revealed, the outcome of text message intervention did not significantly differ between the control and t intervention groups. ^20 - 22^ It can explain that text messages increase the participants’ knowledge but do not empower them with skills building, the most necessary aspect of a behavior change program. ^23^ Although psychosocial support is provided through text messages, it is a one-way communication style. Therefore, a low impact can occur. Thus, a text message might not be considered a stand-alone strategy for behavior change but can be integrated as a tool with another model to be used for the participants. ^24^

Moreover, a study of text messages to improve prenatal health for pregnant women showed that the participants’ health behavior increased significantly different from pre- and post-test. ^25^ The study demonstrates that the theory-based text message can be used to enhance its effectiveness. They used the combination of the theory of Planned Behavior and the technology acceptance model. It can be concluded from the systematic review that the intervention needs to follow the theoretical model of behavior change to increase the effectiveness of text messages. ^26^ Still, the present study did not follow it; all the text messages were information-based following physiological changes during pregnancy. Furthermore, another study evaluated both the dosage and content of text messages. ^27^ The results showed that motivation messages were more effective than information messages for increasing specific behaviors. A review of changing behavior in pregnant women revealed that the empowerment dimension on skills and competencies could lead to success in behavior change programs. ^23^ The present study developed the message based on an educational message and did not empower the participants to follow it. This might be the reason for the non-significant difference between the intervention and control groups in the domains. 

On the other hand, the present study developed messages in many topics throughout the pregnancy period and lack of a specific, clear outcome as other text messages studied. The intervention group who received text messages to improve antenatal care visit had a higher odds ratio for achieving four or more antenatal care visits than the control group. ^28 , 29^ Thus, the lack of a specific outcome can reduce the efficacy of the intervention. ^25^

Furthermore, the study results revealed that the total score of STAI-S in the control group was higher than the intervention group. It seems that the participants who received text messages had less anxiety than those who did not. It can explain that text messages could be used as the prenatal support in the antenatal period since they clarify the maternal changes, health care behavior, signs, and symptoms management throughout the prenatal period. This support through text messages is one of the key factors that reduce anxiety and stress during pregnancy. The support mechanisms assist in improving adaptation and emotions. The study of social support and anxiety level during pregnancy showed that it could reduce psychological problems created by stressful experiences when social support increased. ^30^

One of the strengths of this study was the validated content of the text messages to ensure that pregnant women received quality information. Secondly, the content of text messages covered all appropriate health care points throughout pregnancy, for 13 weeks to 40 weeks, such as nutrition, physical activity, health responsibility, and symptoms management, especially minor discomfort. Thirdly, text messages can relieve the anxiety of the intervention group as a supportive tool for participants during the pregnancy period. However, this study had some limitations. First, the small sample size might decrease the statistical power of the study results. The researcher tried to increase this power by adding a dose-response via text messages throughout a long period between 13 and 40 gestational weeks. Despite expanding the intervention time, the research did not significantly differ in health care behaviors between the groups, except physical activity. Second, this text message intervention only provided content-based knowledge to the pregnant women; it did not develop skills or performance. Thirdly, this study lacks a theoretically driven intervention necessary to succeed; thus, the research needs to include the theory-based behavior change in the study. Fourthly, only educational messages are not enough; the motivational messages should add to persuade and empower the participants to change their behavior. Finally, the study should specify a single health issue, better than many issues applied in this study.

## CONCLUSION

Text messages could increase physical activity in Thai pregnant women and reduce the total score of anxiety during pregnancy. Thus, the text message strategy is appropriate to be used during the antenatal period. Further intervention studies are recommended to be conducted using the theory-based approach to increase skill-building and improve performance via the motivation and empowering message and a single health issue to change the participants’ health behaviors.

## ACKNOWLEDGEMENT

This study was supported by a grant from the Faculty of Nursing, Chiang Mai University. The authors thank Professor Yogo Narita, Mie University, Japan, for his valuable help. 

**Conflict of Interest:** None is declared. 

## References

[1] Coleman J, Eriksen J, Black V, et al ( 2020). The mobile alliance for maternal action text message-based mHealth intervention for maternal care in South Africa: Qualitative user study. JMIR Human Factors.

[2] Colaci D, Chaudhri S, Vasan A ( 2016). mHealth interventions in low-income countries to address maternal health: A systematic review. Annals of Global Health.

[3] Head KJ, Noar SM, Iannarino NT, Grant Harrington  N ( 2013). Efficacy of text messaging based interventions for health promotion: A meta-analysis. Social Science and Medicine.

[4] Poorman E, Gazmararian M, Parker RM, et al ( 2015). Use of text messaging for maternal and infant health: A systematic review of the literature. Maternal and Child Health Journal.

[5] Sondaal SFV, Browne JL, Amoakoh-Coleman M, et al ( 2016). Assessing the effect of mHealth interventions in improving maternal and neonatal care in low- and middle-income countries: A Systematic Review. PLoS One.

[6] Abraha YG, Gebrie SA, Garoma DA, et al ( 2017). Effect of mHealth in improving antenatal care utilization and skilled birth attendance in low-and middle-income countries: a systematic review protocol. JBI Database of Systematic Reviews and Implementation Reports.

[7] Feroz A, Perveen S, Aftab W ( 2017). Role of mHealth applications for improving antenatal and postnatal care in low and middle income countries: a systematic review. BMC Health Services Research.

[8] Coomes CM, Lewis MA, Uhrig JD, et al ( 2012). Beyond reminders: a conceptual framework for using short message service to promote prevention and improve healthcare quality and clinical outcomes for people living with HIV. AIDS Care.

[9] Butchon R, Liabsuetrakul T, Bumpenboon T, Teerawattananon Y ( 2019). Anxiety at first and subsequent pregnancies and its associated factors: A historical cohort study from northeastern Thailand. Journal of Health Science and Medical Research.

[10] Grigoriadis S, Graves L, Peer M, et al ( 2018). Maternal anxiety during pregnancy and the association with adverse perinatal outcome: Systematic review and meta-analysis. Journal of Clinical Psychiatry.

[11] Staneva A, Bogossian F, Pritchard M, Wittkowski A ( 2015). The effects of maternal depression, anxiety, and perceived stress during pregnancy on preterm birth: a systematic review. Women and Birth.

[12] Lagadec N, Steinecker M, Kapassi A, et al ( 2018). Factors influencing the quality of life of pregnant women: a systematic review. BMC Pregnancy and Childbirth.

[13] Shahhosseini Z, Pourasghar M, Khalilian A, Salehi F ( 2015). A review of the effects of anxiety during pregnancy on children’s health. Materia Socio-medica.

[14] Jareethum R, Titapant V, Chantra T, et al ( 2008). Satisfaction of healthy pregnant women receiving short message service via mobile phone for prenatal support: A randomized controlled trial. Journal of the Medical Association Thailand.

[15] Zhong B ( 2009). How to calculate sample size in randomized controlled trial?. Journal of Thoracic Disease.

[16] Spielberger CD, Gorsuch RL, Lushene RE (1970). Manual for the state-trait anxiety inventory.

[17] Delgado AM, Freire AB, Wanderley ELS, Lemos A ( 2016). Analysis of the construct validity and internal consistency of the state-trait anxiety inventory (STAI) state-anxiety (S-Anxiety) scale for pregnant women during labor. Revista Brasileira de Ginecologia e Obstetrícia.

[18] Wongpakaran N, Wongpakaran T ( 2010). The Thai version of the PSS-10: An investigation of its psychometric properties. BioPsychoSocial Medicine.

[19] American College of Obstetricians and Gynecologists ( 2013). ACOG Committee opinion no. 548: weight gain during pregnancy. Obstetrics and Gynecology.

[20] Abroms LC, Johnson PR, Heminger CL, et al ( 2015). Quit4baby: results from a pilot test of a mobile smoking cessation program for pregnant women. JMIR Mhealth and Uhealth.

[21] Huberty JL, Buman MP, Leiferman JA, et al ( 2017). Dose and timing of text messages for increasing physical activity among pregnancy women: a randomized controlled trial. Translational Behavioral Medicine.

[22] Abroms LC, Johnson PR, Leavitt LE, et al ( 2017). A randomized trial of text messaging for smoking cessation in pregnant women. American Journal of Preventive Medicine.

[23] Zinsser LA, Stoll K, Wieber F, et al ( 2020). Changing behaviour in pregnant women: A scoping review. Midwifery.

[24] Cole-Lewis H, Kershaw T ( 2010). Text messaging as a tool for behavior change in disease prevention and management. Epidemiologic Reviews.

[25] Blackwell TM, Dill LJ, Hoepner LA, Geer LA ( 2020). Using text messaging to impreove access to prenatal health information in urban African American and Afro-Caribbean immigrant pregnant women: Mixed methods analysis of text4Baby usage. JMIR MHealth and UHealth.

[26] Poorman E, Gazmararian J, Parker RM, et al ( 2015). Use of text messaging for maternal and infant health: a systematic review of the literature. Maternal and Child Health Journal.

[27] DeTolly K, Skinner D, Nembaware V, Benjamin P ( 2012). Investigate into the use of short message services to expand uptake of human immunodeficiency virus testing and whether content and dosage have impact. Telemedicine Journal and E-health.

[28] Lund S, Nielsen BB, Hemed M ( BMC Pregnancy Childbirth 2014). et al. Mobile phones improve antenatal care attendance in Zanzibar: a cluster randomized controlled trial.

[29] Coleman J, Black V, Thorson AE, Eriksen J ( 2020). Evaluating the effect of maternal mHealth text messages on uptake of maternal and child health care services in SouthAfrica: a multicentre cohort intervention study. Reproductive Health.

[30] Duman, NB, Kocak C ( 2013). The effect of social support on state anxiety levels during pregnancy. Social Behavior and Personality Journal.

